# Enhancement of Storage Stability and Masking Effect of Curcumin by Turmeric Extract-Loaded Nanoemulsion and Water-Soluble Chitosan Coating

**DOI:** 10.3390/pharmaceutics14081547

**Published:** 2022-07-25

**Authors:** Bom Nae Lee, Su Jung Hong, Mi Hee Yu, Gye Hwa Shin, Jun Tae Kim

**Affiliations:** 1 Department of Food Science and Technology, Keimyung University, Daegu 42601, Korea; bomnae1110@gmail.com; 2 Department of Food and Nutrition, BioNanocomposite Research Center, Kyung Hee University, Seoul 02447, Korea; tnwjd0799@naver.com; 3 Research Institute of Biomedical Engineering, Department of Cell Biology, Catholic University of Daegu School of Medicine, Daegu 42472, Korea; miheeyu555@gmail.com; 4 Department of Food and Nutrition, Kunsan National University, Gunsan 54150, Korea; winnie19@kunsan.ac.kr

**Keywords:** turmeric extract, nanoemulsion, water-soluble chitosan, stability, masking effect

## Abstract

This study focused on improving curcumin stability in various pHs and NaCl concentrations and reducing the strong scent of turmeric by the nanoemulsions system and further coating with water-soluble chitosan (WSC). Turmeric extract-loaded nanoemulsions (TE-NEs) were firstly prepared by mixing an oil phase containing turmeric extract, MCT oil, and lecithin, and an aqueous phase containing tween 80 using an ultrasonication method. TE-NEs were further coated with WSC in the ratio of TE-NEs and WSC (1:1 to 1:10). The optimum WSC-TE-NEs exhibited an average particle size of 182 nm, a PDI of 0.317, and a zeta potential of +30.42 mV when WSC-TE-NEs were prepared in the ratio of 1:1. The stability of the WSC-TE-NEs was also assessed by determining the remained curcumin content. The remained curcumin contents of the TE-NEs and the WSC-TE-NEs were higher than that of the turmeric extract (TE) at pH 2~7 and NaCl concentrations of 100~400 mM. Fourier transform infrared (FT-IR) spectra, transmission electron microscope (TEM), and confocal laser scanning microscope (CLSM) images confirmed that the TE-NEs were successfully encapsulated with a WSC coating. As a result of GC analysis, the content of aromatic-turmerone was significantly decreased in the TE-NEs and the WSC-TE-NEs compared to the pristine TE, but there was no significant difference between the TE-NEs and the WSC-TE-NEs. These results suggest that water-soluble chitosan-coated nanoemulsions may be suitable for improving the chemical stability and masking effect of curcumin to facilitate its application in food.

## 1. Introduction

The functional components of turmeric (*Curcuma longa* L.) are known as curcuminoids which are the heavy yellow-brown fraction of turmeric. Curcuminoids are made up of curcumin and its analogs, demethoxycurcumin (DMC), and bis-demethoxycurcumin (BDMC). Curcuminoids have shown potent antioxidant, anticancer, antibacterial, and antiviral activities [1,2]. Most research on the encapsulation of curcuminoids has focused on the development of a carrier system for curcumin, which is the main active compound in turmeric [3,4,5]. Nevertheless, curcumin accounts for less than 5% of the total turmeric mass, and because of the high cost of purification, turmeric extract will be preferred for the preparation of nanoemulsions for commercialization [6,7]. Curcumin is generally recognized as safe (GRAS) by the United States Food and Drug Administration (U.S. FDA) due to its low toxicity even when it was consumed at relatively high levels [8]. The effectiveness of curcumin as a nutraceutical or natural colorant in food depends on its ease of use, chemical stability, and bioavailability. However, the exposure to light, heat, and oxygen during the traditional extraction process of turmeric will degrade the curcuminoids, reduce yields, and limit its application to food [9,10,11]. Moreover, turmeric and curcumin are poorly absorbed and have very low bioavailability. Thus, various nanoformulations have been developed to improve the absorption and bioavailability of curcumin. Nanoemulsion is a good candidate for the commercial applications of turmeric and curcumin. Nano-scale delivery systems may confer curcumin with rapid dissolution rates, higher stability, tailored release patterns, higher permeability, higher bioavailability, and many other advantages compared with micro-scale or larger delivery systems [12,13].

In addition, various nano-scale delivery systems have been evaluated to encapsulate turmeric extract and integrate it into food and beverage systems [7]. However, the inherent pungency of turmeric extract can affect the overall organoleptic desirability of the food to which it is applied. Therefore, masking the turmeric aroma is a fundamental component of the widespread use of turmeric extracts in the food industry [14].

Encapsulation is one of the most effective solutions to mask the unpleasant odor and smell and protect the sensitive bioactive agents through entrapment in a carrier system. Thus, this technology protects active compounds against evaporation, reaction, or migration, masks odors, stabilizes food ingredients, and increases their bioavailability [15]. Natural polymers such as chitosan, alginate, agarose, carrageenan, and pectin have been popularly used materials to encapsulate functional compounds. Biopolymers are generally used for coating nano-delivery systems to increase their stability and adsorption rate and to modulate the payload release pattern [16]. Chitosan is an interesting natural polysaccharide as a wall material of functional ingredients due to its non-toxic, biodegradable, biocompatible, and positively charged properties. Chitosan has been popularly used as a coating material for various delivery systems and showed improvement in adsorption and mucoadhesion properties by strong electrostatic interactions between negatively charged mucin and positively charged chitosan molecules [3]. However, the use of organic acids to dissolve chitosan has been limited in its applications in the food industry.

The ultimate objective of this study is to improve the stability of turmeric extract-loaded nanoemulsions (TE-NEs) by a water-soluble chitosan (WSC) coating and to enhance the masking effect of aromatic turmerone in turmeric extract by a nanoemulsion system and further WSC coating. For this, turmeric extract-loaded nanoemulsions (TE-NEs) were firstly prepared by an ultrasonication method. Then, water-soluble chitosan was used to make a coating of the prepared TE-NEs, and its effects on the colloidal stability and storage stability of the TE-NEs under environmental conditions such as different pHs, ionic strengths, and temperatures were investigated by measuring the particle size, zeta potential, and the remained curcumin content of TE-NEs.

## 2. Materials and Methods

### 2.1. Materials

Turmeric extract powder (curcumin content of 12.45 mg/g) was kindly provided by Ottogi Corporation (Anyang, Korea). Curcumin (≥98%) and aromatic turmerone were purchased from Sigma-Aldrich (St. Louis, MO, USA). MCT (medium-chain triglyceride) oil was purchased from NOW Foods (Elmhurst, IL, USA). Soy lecithin and tween 80 were purchased from ES Food (Gunpo, Korea) and Daejung Chemical (Seoul, Korea). Water-soluble chitosan (MW: 842 Da, DOD: 93.54%) was kindly provided by YangYang Chitosan Company (Sokcho, Korea).

### 2.2. Preparation of Turmeric Extract-Loaded Nanoemulsions (TE-NEs)

Turmeric extract-loaded nanoemulsions (TE-NEs) were prepared by our previous method [6,9]. Briefly, the oil phase was prepared by dissolving turmeric extract powder (TE) in MCT oil and adding soy lecithin. The aqueous phase was prepared by mixing tween 80 and distilled water. The ratio of oil, surfactant, and water is shown in Table 1. First, the oil phase and aqueous phase were mixed using a magnetic stirrer and homogenized by a high-speed homogenizer (HSH) (HG-15D, Daihan Scientific Co., Ltd., Wonju, Korea) at 5000 rpm for 10 min. Then, turmeric extract-loaded nanoemulsions (TE-NEs) were prepared by ultrasonication (US) with a Vibra Cell (VCX-750, Sonics & Materials, Inc., Sandy Hook, Newtown, CT, USA) at 40% amplitudes for 15 min.

### 2.3. Preparation of Water-Soluble Chitosan-Coated TE-NEs (WCS-TE-NEs)

To prepare the water-soluble chitosan-coated TE-NEs (WCS-TE-NEs), the TE-NE solution was drop-wised to the same volume of 2.5% (*w/v*) water-soluble chitosan solution under agitation using a peristaltic pump (BT-300CA, Baoding Lead Fluid Technology Co., Ltd., Baoding, China) at a flow rate of 500 µL/min. The mixture was magnetically stirred at room temperature under 300 rpm for 2 h to obtain the WCS-TE-NEs. Then, the WCS-TE-NEs were centrifuged at 10,000 rpm for 30 min and the supernatant was collected for further analysis. The solution of WCS-TE-NEs was stored at 4 °C until it was characterized.

### 2.4. Droplet Size, Polydispersity Index, and Zeta Potential

The mean droplet size, polydispersity index (PDI), and zeta potential of the TE-NEs and WCS-TE-NEs were measured by dynamic light scattering (DLS) using a zeta potential and particle size analyzer (ELSZ-2000, Otsuka Electronics, Tokyo, Japan). Briefly, 1 mL of TE-NEs and WCS-TE-NEs dispersion was added to a polystyrene latex cell, and their droplet size, PDI, and zeta potential were measured at 25 °C with a detector angle of 90° and wavelength of 633 nm. Each sample was measured in triplicate and the average values were used.

### 2.5. Encapsulation Efficiency

The encapsulation efficiency (EE) of the WCS-TE-NEs was determined by HPLC (D-20A, Shimadzu, Kyoto, Japan) analysis. The WCS-TE-NEs dispersion was centrifuged at 10,000 rpm for 30 min to separate the WCS-TE-NEs in the precipitate and the supernatant; the latter one was stirred in hexane at 25 °C for 2 h to extract the unencapsulated curcumin. The obtained supernatant was mixed with methanol in a ratio of 1:9 (*v/v*) and analyzed by HPLC to determine the free curcumin content. EE was calculated using the following Equation (1):

EE (%) = [(*C_t_* – *C_f_*)/*C_t_*] × 100 (1)

where *C_t_* is the amount of total curcumin in the WCS-TE-NEs and *C_f_* is the amount of free curcumin in the supernatant.

### 2.6. Temperature Stability of the WCS-TE-NEs

The WCS-TE-NEs samples were transferred into glass vials, then they were incubated in a water bath at 4, 25, and 60 °C for 14 days. The particle size and zeta potential of the WCS-TE-NEs were measured every 2 days and their changes were compared for the temperature stability.

### 2.7. Ionic Strength Stability of the WCS-TE-NEs

Ionic strength usually increases during food processing. Hence, it is desirable for the nanoemulsion systems to withstand higher ionic strength with little or no change in curcumin content. NaCl solution was added to the turmeric extract powder (TE), TE-NEs, and WCS-TE-NEs samples to adjust the NaCl concentration to 100~400 mM. Changes in the curcumin contents at various NaCl concentrations were then determined by HPLC analysis.

### 2.8. pH Stability of the WCS-TE-NEs

The TE-NEs and the WCS-TE-NEs were dispersed in an aqueous buffer solution. Then, their pHs were adjusted to 2, 5, 7, and 10 using 1 M NaOH and 1 M HCl solutions. Samples were then transferred into conical tubes and stored at 37 °C for 24 h. After cooling to room temperature, the curcumin contents were measured by HPLC.

### 2.9. FT-IR Spectroscopy

The interactions between the TE-NEs and water-soluble chitosan molecules were investigated by Fourier transform-infrared spectroscopy (FT-IR; NICOLECT iS5, Thermo Fisher Scientific, Madison, WI, USA). All IR spectra were measured at a 4 cm^−1^ resolution using a range of 4000–400 cm^−1^ and a scanning speed of 16 scans/min in transmission mode.

### 2.10. HPLC

A Shimadzu D-20A HPLC (Kyoto, Japan) fitted with a UV absorbance detector (operated at 265 nm) and an ACE5 C18 column (4.6 × 250 mm, 5 µm; Advanced Chromatography Technologies, Aberdeen, UK) was used to determine the amount of curcumin in the samples. The mobile phase was 100% methanol and pumped at a flow rate of 1.0 mL/min. A sample volume of 20 µL was injected.

### 2.11. Gas Chromatography

Known amounts of TE, TE-NEs, and WCS-TE-NEs were added to 20 mL headspace (HS) vials and sealed with PTFE/silicone septa. The vials were then incubated at 70 °C for 10 min. Afterward, 1 mL of vapor was withdrawn and injected directly into the chromatographic column via a transfer line. The volatile composition was analyzed using an Agilent 7890A series gas chromatograph (Agilent Technologies, Santa Clara, CA, USA) fitted with an HP-1 column (30 m × 0.25 mm × 0.25 µm film thickness, Agilent Technologies). Nitrogen gas was used as a carrier gas at a flow rate of 1 mL/min. Samples (1 µL) were injected with a split ratio of 30:1. The chromatographic run was started by warming the column at 70 °C for 1 min, heated to 280 °C at a rate of 5 °C/min, and held at 250 °C for 5 min. The compounds were identified by comparison with authentic standards. Quantification of volatile components was performed by comparing the sample peak area to a known amount of pure components.

### 2.12. Transmission Electron Microscopy

The morphological characteristics of TE-NEs and WCS-TE-NEs were determined by transmission electron microscopy (TEM, HT 7700, Hitachi, Ltd., Tokyo, Japan). The TE-NEs and WCS-TE-NEs were diluted 10 times, and a drop of the diluted solution was applied to a carbon-coated copper (Cu) grid (300 mesh). The grid was kept under ambient conditions for 30 s. This step was repeated three times. As a negative staining agent, phosphotungstic acid was applied to the grid for 1 min, and the sample was dried overnight and imaged.

### 2.13. Confocal Laser Scanning Microscopy

Confocal laser scanning microscopy (CLSM) was used to observe the morphology and deposition mechanism of TE-NEs and WCS-TE-NEs. Briefly, 3 µL of Nile Red O fluorescent dye solution (0.02%, *w/v*) was added to 500 µL of the TE-NEs to stain the MCT oil. As an amine-reactive fluorescent dye, fluorescein isothiocyanate (FITC) was used to stain WSC. The synthesis of FITC-labeled WSC was based on the reaction between isothiocyanate group of FITC and the amino group of chitosan [17]. WSC was stained by mixing WSC with 0.05% (*w/v*) FITC solution in the dark at ambient temperature for 4 h. The FITC-labeled WSC was precipitated by centrifuge at 10,000 rpm for 30 min and the unreacted FITC was separated until no fluorescence was detected in the supernatant. CLSM 700 confocal microscope (Carl Zeiss, Oberkochen, Germany) was used to analyze the samples at a magnification of 40×. Nile Red O and FITC dyes were excited at 543 and 488 nm, respectively. The z-stack images were obtained using the LSM 700 ZEN software.

### 2.14. Statistical Analysis

Droplet size, zeta potential, and encapsulation efficiency of TE-NEs and WCS-TE-NEs were statistically analyzed using one-way analysis of variance (ANOVA). The Statistical Package for the Social Science (SPSS, version 20.0, SPSS Inc., Chicago, IL, USA) was used for the analysis. Duncan’s multiple range tests were used to determine any significant difference at *p* < 0.05 in each group.

## 3. Results

### 3.1. Characterization of TE-NEs and WCS-TE-NEs

**Figure 1:**
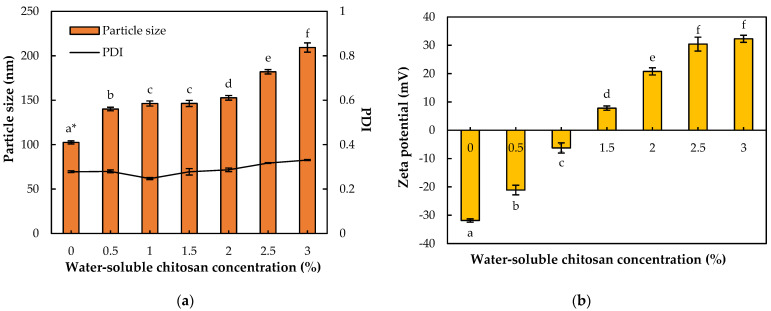
Effect of water-soluble chitosan (WSC) concentration on the physicochemical properties of the WCS-TE-NEs. Each WSC solution was mixed to TE-NE solution in ratio of 1:1 (*w/w*). (**a**) Particle size and PDI; (**b**) zeta potential of the WCS-TE-NEs. * Different letters indicate significant differences at *p* < 0.05 by Duncan’s multiple range test.

MCT oil was selected as an oil phase to dissolve turmeric extract powder (TE) because TE showed the highest solubility (0.696 mg/g) in MCT oil among the most commonly used oil types in our previous study [6]. Both the TE-NEs and the WCS-TE-NEs showed high stability at 4 °C storage for 15 days without any phase separation. As indicated earlier, the droplet size, PDI, and zeta potential of the TE-NEs before WCS coating were about 102.5 nm, 0.278, and −31.9 mV, respectively. As shown in Figure 1a, the droplet size of the WCS-TE-NEs was significantly (*p* < 0.05) increased to 140.1 nm with a 0.5% (*w/v*) WCS coating and further increased to 209.3 nm with 3% (*w/v*) WCS coating. It is common that the droplet size of the nanoemulsions increased by coating with biopolymers [18]. The PDI of the WCS-TE-NEs were slightly increased from 0.278 to 0.331 as the WCS concentration increased, but tended to remain below 0.3, except for that of the WCS-TE-NEs with 3.0% WCS coating. It means that the particle size of each WCS-TE-NE is evenly distributed and its size distribution is narrow.

The zeta potential of the WCS-TE-NEs ranged from –31.87 mV for the control TE-NEs to +32.29 mV for the TE-NEs with a 3% (*w/v*) WCS coating (Figure 1b). Increasing the WCS concentration resulted in the progressive decrease of the negative charge of the droplets. As a result, the electrostatic repulsion between the droplets was not sufficiently large to prevent droplet aggregation. It has been estimated that a minimum zeta potential value of ±30 mV is required to generate an electrostatic repulsion strong enough to overcome the attractive interactions such as van der Waals force and hydrophobic force [19]. When the WCS concentration was slightly higher than 1% (*w/v*), the droplet charge became almost zero (neutral). Furthermore, this outcome also implied that the droplet surfaces had become saturated with WSC molecules. This phenomenon indicated that the cationic chitosan was adsorbed and self-assembled onto the surface of the negatively charged lipid droplets, thus forming a layer around the droplets with a net positive charge [20].

### 3.2. Effect of the Ratio of TE-NEs to WCS Solution on the Particles Size, PDI, and Zeta Potential

**Figure 2:**
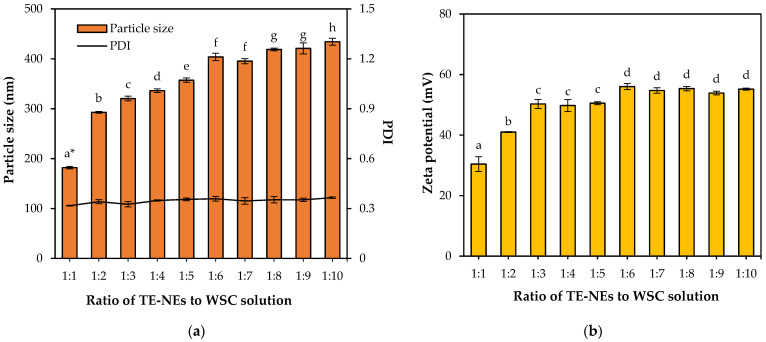
Effect of ratio of nanoemulsion to water-soluble chitosan on the physicochemical properties of the WCS-TE-NEs. (**a**) Particle size and PDI; (**b**) zeta potential of the WCS-TE-NEs. * Different letters indicate significant differences at *p* < 0.05 by Duncan’s multiple range test.

WSC concentrations were varied from 0.5% to 3.0% and 2.5% WSC solution was selected as a proper coating material which obtained a narrow size distribution with less than 0.3 of PDI and high stability with over +30 mV of zeta potential. The ratios of TE-NEs to WCS were varied from 1:1 to 1:10. The particle size of the WCS-TE-NEs were significantly (*p* < 0.05) increased from 182.0 nm to 434.3 nm with an increasing WSC concentration, as shown in Figure 2a. The minimum particle size of the WCS-TE-NEs was 182.0 nm, obtained when the ratio of TE-NEs to WSC was 1:1. Polydispersity index (PDI) values < 0.3 mean that the sample has a highly monodisperse and uniform state, whereas PDI values > 0.7 indicate that the sample has a wide size distribution and is probably not suitable for food applications. The PDI of the WCS-TE-NEs were ranged between 0.317 and 0.366, indicating that the nanoemulsion was in a relatively uniform state. Therefore, when the ratio of TE-NEs to WSC is 1:1, a small diameter and stable emulsion is obtained. The zeta potential of the WCS-TE-NEs ranged from +30.42 mV to +55.18 mV when the ratio of TE-NEs to WSC was 1:1 to 1:10, as shown in Figure 2b. In TE-NEs and WSC ratios of 1:1 to 1:6, the zeta potential of the WCS-TE-NEs significantly (*p* < 0.05) increased, but there was no significant (*p* > 0.05) difference above 1:6. In all cases, the zeta potential was +30 mV, indicating that they are resistant to aggregation and coalescence.

### 3.3. Effect of the Ratio of TE-NEs to WCS Solution on the Encapsulation Efficiency

**Figure 3:**
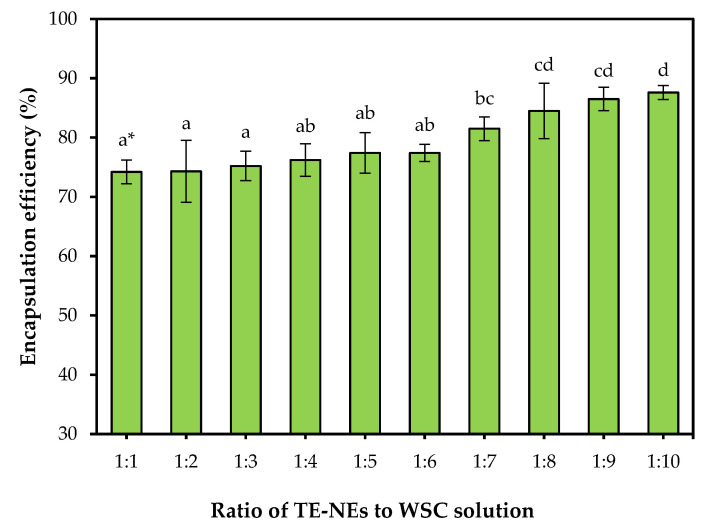
Effect of the TE-NEs/chitosan ratio on the encapsulation efficiency of the WCS-TE-NEs. * Different letters indicate significant differences at *p* < 0.05 by Duncan’s multiple range test.

To determine the most suitable ratio of TE-NEs to WSC, we investigated the encapsulation efficiency of the WCS-TE-NEs while increasing the amount of WSC solution. Figure 3 shows the effect of varying the ratio of TE-NEs to WSC on the encapsulation efficiency of the WCS-TE-NEs. The encapsulation efficiency of the WCS-TE-NEs were increased from 74.2% to 87.6% as the proportion of WSC increased (1:1 to 1:10). The ratio of TE-NEs to WSC clearly affected the encapsulation efficiency of the WCS-TE-NEs. Hong et al. [21] showed a similar trend of increasing the encapsulation efficiency of iron from iron-solid lipid nanoparticles by water-soluble chitosan coating, but their encapsulation efficiency was ranged from 80.61% to 97.58%. This is because of the different formulations showed different encapsulation efficiencies and solid lipid nanoparticles showed a higher encapsulation efficiency than the emulsion system. Although the 1:1 ratio formulation had the lowest encapsulation efficiency, it was chosen for further experiments considering the results of particle size, PDI, and zeta potential values.

### 3.4. Effect of the Storage Temperature on the Particle Size and Zeta Potential

**Figure 4:**
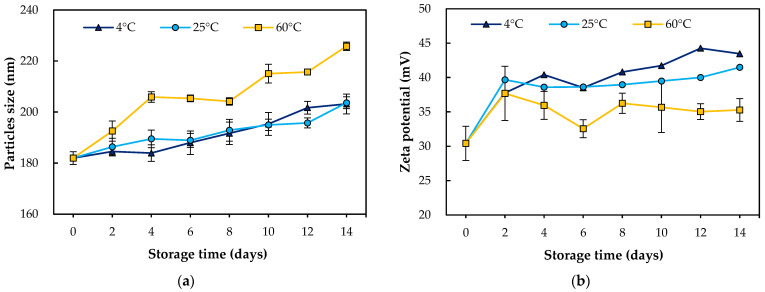
Effect of temperature on the physicochemical properties of the WCS-TE-NEs. (**a**) Particle size; (**b**) zeta potential of the WCS-TE-NEs.

The stability of the WCS-TE-NEs was evaluated by measuring the changes in the particle size and zeta potential over storage time. The WCS-TE-NEs were stored at 4 °C, 25 °C, and 60 °C for up to 14 days, and their particle size and zeta potential were measured by DLS at 0, 2, 4, 6, 8, 10, 12, and 14 days (Figure 4). The particle size of the WCS-TE-NEs were significantly (*p* < 0.05) increased at all temperatures during storage. The bigger particle size of the WCS-TE-NEs at 14 days may be mainly ascribed to their swelling over storage time [3]. The particle size of the WCS-TE-NEs were steadily increased at 4 °C and 25 °C for 14 days, but the increase was much lower than that of 60 °C. The PDI of the WCS-TE-NEs was significantly (*p* < 0.05) decreased at 60 °C during storage. The zeta potential is considered to be an important parameter to assess emulsion stability as it governs the degree of repulsion between similarly charged dispersed droplets [22]. The zeta potentials of the WCS-TE-NEs were significantly (*p* < 0.05) increased from +30.4 mV to +43.5 mV at all temperatures. As expected, the WCS-TE-NEs were positively charged because of the positively charged amino groups of chitosan [23].

### 3.5. Effect of the NaCl Concentration and pH on the Content of Remained Curcumin

**Figure 5:**
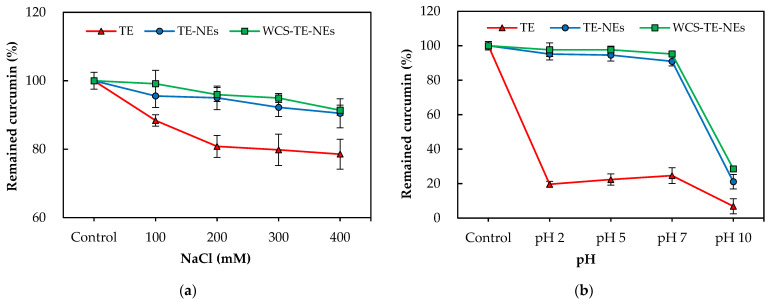
Effect of NaCl concentrations (**a**); pH changes (**b**) on the physicochemical properties of the TE, TE-NEs, and WCS-TE-NEs.

In order to discover the potential applications of nanoemulsions to foods, the influence of the NaCl levels and pH conditions on the curcumin content was measured. Nanoemulsions may be used in food and beverage products with different salt levels, and therefore, it is important to establish the impact of ionic strength on droplet stability. The stability of the emulsion plays an important role on the stability of curcumin, since the oil–water interface prevents the curcumin from being in direct contact with the oxygen and free radicals in the water [24]. The retention rate of curcumin in the presence of NaCl (0–400 mM) decreased from 100% to 78.5% upon the addition of NaCl to the TE. However, the concentration of curcumin decreased from 100% to 90.5% in the TE-NEs, and from 100% to 91.4% in the WCS-TE-NEs (Figure 5a). This result suggests that the combination of the repulsive interactions between the chitosan-covered droplets was strong enough to overcome the combination of attractive interactions. Li et al. [18] also reported that the chitosan coating prevented nanoemulsion phase separation and improved storage stability by the ionic strength test.

Figure 5b shows the results of the stability test of TE, TE-NEs, and WCS-TE-NEs subjected to pHs between 2 and 10. Food products and beverages present a wide range of pHs, from acidic beverages such as soft drinks or juices to neutral beverages such as milk or milk substitutes. Therefore, the influence of the pH on curcumin loss was studied. The TE-NEs and the WCS-TE-NEs exhibited better stability than the TE for the pH range of 2–7. However, at pH 10, degradation of curcumin occurred in the TE, TE-NEs, and WCS-TE-NEs. Curcumin degraded rapidly both in acidic as well as neutral conditions. More than 70% of the curcumin degraded in basic conditions, while 40–60% degradation occurred under acidic pH conditions. Curcumin is unstable in an alkaline medium because it is exposed to hydrolytic degradations. Under these conditions, the yellow color of curcumin turns red [25]. These results are consistent with those of previous studies, which have also reported that emulsion system itself retarded the curcumin degradation at acidic and alkaline pHs compared to native curcumin [9,26].

### 3.6. Physical Interaction of TE-NEs with Water-Soluble Chitosan

**Figure 6:**
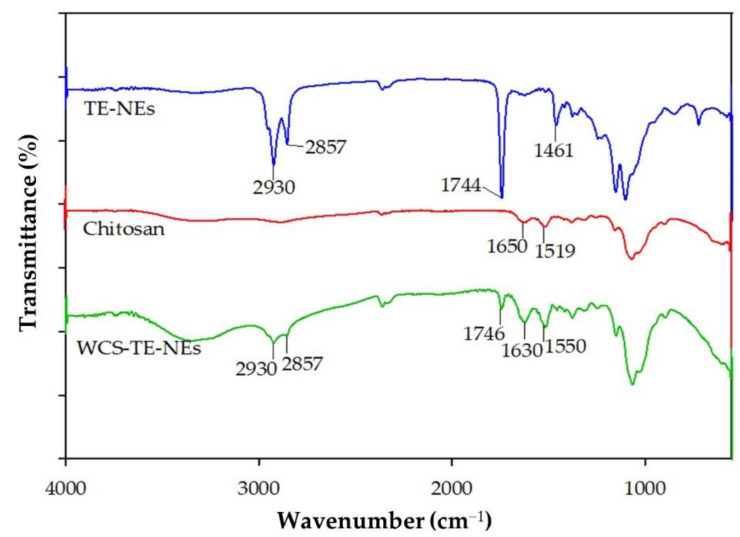
FT-IR spectra of TE-NEs, water-soluble chitosan, and WCS-TE-NEs.

The chemical structure of the adsorbent is of vital importance in understanding the adsorption process. The FT-IR technique is an important tool used to identify the characteristic functional groups that are instrumental in the adsorption of aromatic compounds [27]. Figure 6 shows the FT-IR spectra of the TE-NEs, chitosan, and the WCS-TE-NEs. Four characteristic peaks at 2930, 2857, 1744, and 1461 cm^−1^ were observed in the TE-NEs, which were assigned to C-H banding, CH_2_ symmetrical deformation, C=O stretching, and CH_3_ symmetrical deformation, respectively. Two peaks at 1650 and 1519 cm^−1^ were observed in native chitosan, which were assigned to the carbonyl (C=O) stretching (amide I) and N-H bending vibration (amide II), respectively [28,29]. The FT-IR spectrum of the WCS-TE-NEs showed the combination of those of the TE-NEs and chitosan. These results supported that the TE-NEs droplets were successfully coated by the chitosan solution.

### 3.7. Effect of Encapsulation on Flavor Components

**Figure 7:**
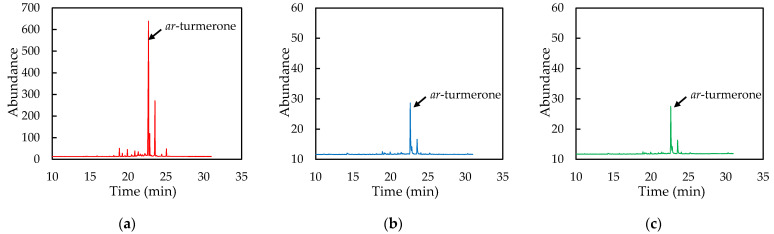
Gas chromatograms of ar-turmerone in TE (**a**), TE-NEs (**b**), and WCS-TE-NEs (**c**).

The gas chromatograms are shown in Figure 7. Aromatic turmerone (ar-turmerone) was reported as a major component in the essential oil of turmeric [2]; thus, we focused on the presence of this compound in the turmeric headspace samples. The amount of ar-turmerone was calculated as the peak area. The ar-turmerone peak areas of the TE, TE-NEs, and WCS-TE-NEs were 3429, 74, and 72, respectively. Hence, compared with the TE, both the TE-NEs and the WCS-TE-NEs showed significantly (*p* < 0.05) reduced peak areas, but there is no significant (*p* > 0.05) difference in between the TE-NEs and the WCS-TE-NEs’ areas.

### 3.8. Morphology by Transmission Electron Microscopy

**Figure 8:**
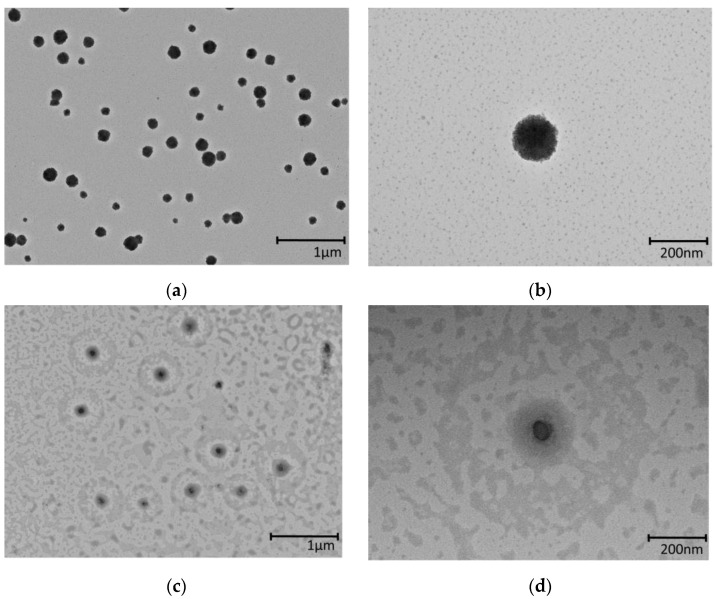
TEM images of TE-NEs (**a**,**b**) and WCS-TE-NEs (**c**,**d**) at a magnification of ×7000 for (**a**,**c**) and ×30,000 for (**b**,**d**).

The surface morphologies of the TE-NEs and the WCS-TE-NEs were characterized by transmission electron microscopy (TEM). Figure 8 shows the TEM images of the TE-NEs and the WCS-TE-NEs particles at magnifications of ×7000 and ×30,000. The morphology of the TE-NEs and the WCS-TE-NEs was almost spherical, and the particle size ranged from 80 to 200 nm. An increase in the particle size through coalescence, flocculation, or aggregation was not observed in the TEM images of the TE-NEs and the WCS-TE-NEs. The WCS-TE-NEs had a bright contrasting band surrounding the droplets, as shown in Figure 8d. It can be seen that the coating layer looks hazy compared to the inner layer in which the particles are present. A higher atomic mass or density of inner layer appears darker because it absorbs more electron beams. The interaction of water-soluble chitosan with the surface of the TE-NEs was thus well visualized.

### 3.9. Confocal Analysis of TE-NEs and WCS-TE-NEs

**Figure 9:**
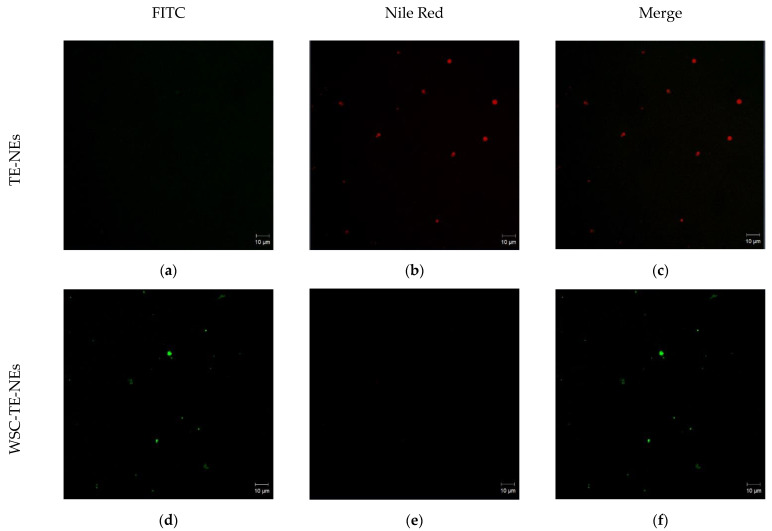
Confocal laser scanning microscopy images of TE-NEs (**a**–**c**) and WCS-TE-NEs (**d**–**f**). Nile Red dye shows a red color by binding with lipids and oils in the TE-NEs, whereas FITC shows a green color by binding with proteins and chitosan in the WCS-TE-NEs.

CLSM microscopy was used to observe the water-soluble chitosan deposition on the nanoemulsion droplets by marking the chitosan molecules and oily core with Nile Red and fluorescein isothiocyanate (FITC), respectively. After excitation with a laser beam, Nile Red dye gives a red color to the lipids and oils, while FITC confers a green color to proteins and chitosan due to the presence of amino groups. A 40× magnification was used to observe the morphology of the polyelectrolyte-coated nanodroplets, and relatively larger (micron size) droplets were selected to clearly observe the deposition mechanism of chitosan onto the oil core [30]. Figure 9 shows the CLSM images of the TE-NEs and the WCS-TE-NEs droplets marked with Nile Red fluorescence dye (red color) and FITC (green color). The chitosan–coated droplets were morphologically spherical. The inner red color represents the turmeric extract oily core, while the green color surrounding the core was identified as a layer of chitosan molecules uniformly deposited on the TE-NEs droplets (core). These results indicate the formation of spherical and stable nano-capsules consisting of TE-NEs and a chitosan-based biopolymer coating.

## 4. Conclusions

The turmeric extract nanoemulsions (TE-NEs) were prepared by ultrasonication, then water-soluble chitosan was used to make a coating of the prepared WSC-TE-NEs. The particle size of the WCS-TE-NEs were between 182.0 nm and 434.3 nm as the ratio of TE-NEs and chitosan solutions were changed from 1:1 to 1:10, and the encapsulation efficiency of the WCS-TE-NEs showed a significant increase from 74.2% to 87.6% as the ratio of chitosan increased. The stability of the TE-NEs and WSC-TE-NEs were tested under various pHs and ionic strengths. As a result, as the concentration of NaCl increased (0–400 mM), the curcumin content of TE decreased by more than 20%, but its content decreased by about 10% in TE-NEs and WSC-TE-NEs. In addition, it was found that TE-NEs and WSC-TE-NEs maintained a higher curcumin content compared to TE in the results of the curcumin content according to pH conditions. Therefore, the TE-NEs and WSC-TE-NEs could be successfully used in food and beverage products.

## Figures and Tables

**Table 1:** Compositions of the turmeric extract-loaded nanoemulsions.

Compositions				
MCT Oil (g)	Surfactants		Distilled Water (g)	Turmeric Extract Powder (g)
	Lecithin (g)	Tween 80 (g)		
7.5	4.1	3.4	85	1.5

## Data Availability

The data presented in this study are available upon request.
